# Integration of Metabolomic and Transcriptomic Provides Insights into Anti-Inflammatory Response to *trans*-10-Hydroxy-2-decenoic Acid on LPS-Stimulated RAW 264.7 Cells

**DOI:** 10.3390/ijms241612666

**Published:** 2023-08-11

**Authors:** Minjie Huang, Jie Dong, Xiaodong Tan, Shuyuan Yang, Minghui Xiao, Deqian Wang

**Affiliations:** Institute of Animal Husbandry and Veterinary Science, Zhejiang Academy of Agricultural Sciences, Hangzhou 310021, China

**Keywords:** 10-HDA, anti-inflammatory, royal jelly, metabolites

## Abstract

*Trans*-10-hydroxy-2-decenoic acid (10-HDA) is a unique fatty acid found in royal jelly that possesses potential health benefits such as anti-inflammatory. However, further research is needed to fully understand its mechanisms of action and therapeutic potential for inflammation-associated diseases. In this present study, liquid chromatography–tandem mass spectrometry (LC-MS/MS) and RNA-seq analyses were conducted to comprehensively analyze the in vitro anti-inflammatory effects of 10-HDA on lipopolysaccharide (LPS)-stimulated RAW 264.7 cells. Our results demonstrated that 128 differentially expressed metabolites and 1721 differentially expressed genes were identified in the 10-HDA-treated groups compared to the LPS groups. Metabolites were significantly enriched in amino acid metabolism pathways, including methionine metabolism, glycine and serine metabolism, and tryptophan metabolism. The differentially expressed genes enrichment analysis indicated that antigen processing and presentation, NOD-like receptor signaling pathway, and arginine biosynthesis were enriched with the administration of 10-had. The correlation analysis revealed that glycerophospholipid metabolism and s-adenosylmethionine-dependent methylation processes might be involved in the response to the 10-HDA treatment. Overall, the findings from this study showed that 10-HDA might involve the modulation of certain signaling pathways involved in the inflammatory response, but further research is needed to determine the safety and efficacy as a therapeutic agent.

## 1. Introduction

Inflammation is a natural process that occurs in response to tissue injury, infection, or other types of damage [1]. However, chronic inflammation is associated with the development of various diseases, including cancer, cardiovascular disease, and autoimmune disorders [2]. Macrophages have been shown to be involved in both the initiation and resolution of inflammation, which switch between a pro-inflammatory (M1) phenotype and an anti-inflammatory (M2) phenotype depending on the signals they receive from their environment [3,4]. Lipopolysaccharide (LPS) has been proven to trigger the production of reactive oxygen species (ROS), which mediates the excessive production of pro-inflammatory cytokines leading to inflammatory response in macrophages [5].

Recent studies have identified various genes and metabolic pathways involved in the regulation of macrophage polarization and inflammation [6,7]. Macrophage activation is tightly regulated by a complex network of signaling pathways involving transcription factors, epigenetic modifications, and post-transcriptional mechanisms [8,9]. The metabolic state of macrophages is determined by the availability of nutrients and the activity of metabolic pathways such as glycolysis, oxidative phosphorylation, and fatty acid metabolism. The dysregulation of these metabolic pathways can lead to altered macrophage activation and contribute to chronic inflammation and metabolic diseases [10]. A study revealed that the AMP-activated protein kinase (AMPK) pathway has been shown to play a role in regulating macrophage polarization and inhibiting inflammation [11]. Therefore, a deeper understanding of these mechanisms may lead to new therapeutic strategies for diseases related to macrophage function.

Royal jelly is a secretion produced by the hypopharyngeal and mandibular glands of worker bees (*Apis mellifera*) with abundant amounts of proteins, free amino acids, lipids, vitamins, and sugars [12]. 10-hydroxy-2-decenoic acid (10-HDA), an unsaturated fatty acid, is the major component of royal jelly, which mainly exists in the royal jelly of the queen bee acid [13]. 10-HDA was proved to inhibit the TLR4-induced immune cell activation [14] and inflammatory cytokine expression in LPS-activated macrophages via reducing NF-κB expression [15]. In our previous study, 10-HDA inhibited nitric oxide (NO) production in a dose-dependent manner, reduced the secretion of TNF-α, IL-1β, and increased the anti-inflammatory cytokine IL-10 in LPS-stimulated RAW 264.7 cells, which indicated that 10-HDA was able to attenuate inflammation and the inflammatory polarization of M1 macrophages [16]. Hence, further understanding and elucidating the molecular mechanisms underlying the anti-inflammatory effects of 10-HDA have potential implications for the promotion of human health. In this present study, we used the liquid chromatography–mass spectrometry (LC-MS) approach to investigate the metabolite profile changes associated with the effects of 10-HDA on the LPS-stimulated RAW 264.7 cells by integrating with transcriptomic data. The data acquired in this study could provide objective support for evaluating the role of 10-HDA as an anti-inflammatory and a molecular basis for further study as a functional additive.

## 2. Results

### 2.1. Intracellular Metabolomic Changes Associated with 10-HDA Treated

**Figure 1:**
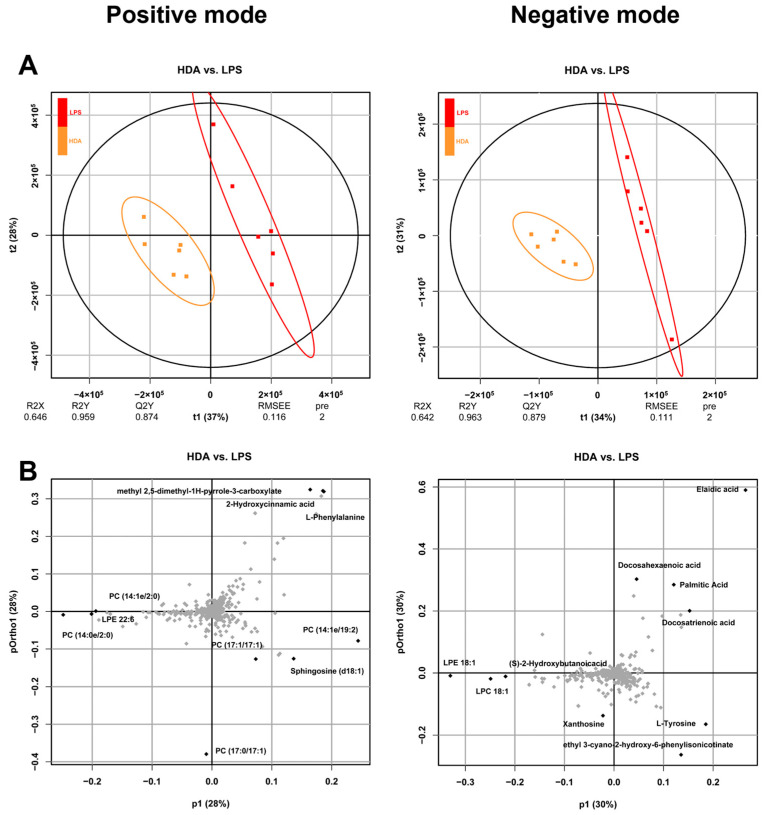
Multivariate analyses of metabolites in RAW 264.7 cells. (**A**) PLS-DA score plots of metabolite profiling data showing different clusters in positive and negative ion mode. Red spots indicate LPS-induced cell groups (LPS), yellow spots indicate 10-HDA-treated cell groups (HDA); (**B**) loading plots representing influential metabolites in the HDA-treated and LPS-stimulated groups under positive and negative ion mode.

To investigate the role of 10-HDA treatment in inflammatory macrophages, LC-MS/MS was performed to analyze the intracellular metabolites in positive and negative ion modes. As shown in Figure 1A, a clear separation between cells from the LPS-treated (LPS) and blank control (BC) group and the LPS and 10-HDA-treated (HDA) group was observed in PLS-DA score plots, which indicated the distinct metabolic properties of different treatments. The metabolites that contribute to the segregation of the HDA group from the LPS group included L-phenylalanine, sphingosine (d18:1), PC (17:1/17:1), and LPE 22:6 in positive ion mode, whereas docosahexaenoic acid, xanthosine and L-tyrosine in negative ion mode (Figure 1B). In total, 112 differential expressed metabolites (DEMs) were detected in LPS vs. BC comparison, including 53 upregulated and 59 downregulated metabolites. A total of 128 differential metabolites were identified in HDA treated as compared with the LPS stimulated cells. Among them, 98 metabolites were upregulated such as PC (16:1e/2:0), PC (18:2e/2:0), and L-glutathione. In addition, 30 metabolites were downregulated, such as methionine, L-tyrosine, and ornithine. These results suggested that 10-HDA treatment led to significant metabolic alterations in LPS-induced macrophages and its potential as a therapeutic agent in inflammatory diseases.

### 2.2. Impacted Metabolic Pathways

**Figure 2:**
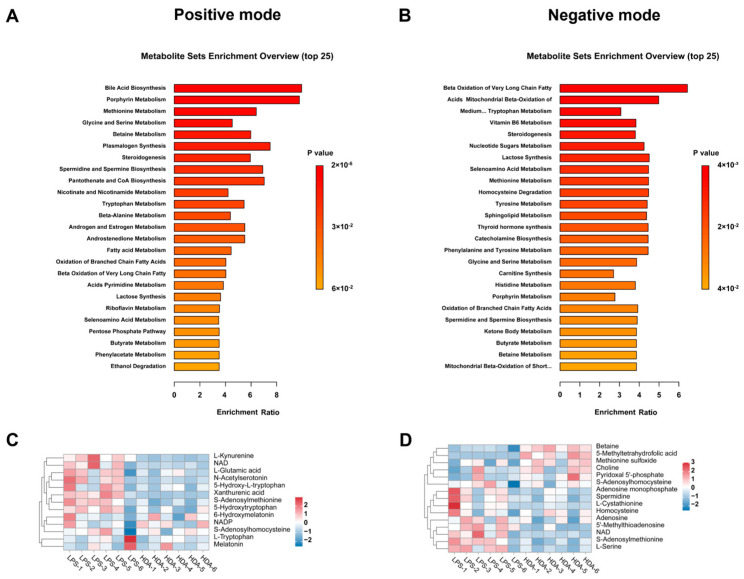
Analyses of HDA-treated group and LPS-stimulated group based on metabolomics. Metabolites set enrichment analysis for pathway over-representation in (**A**) positive and (**B**) negative ion modes. Hierarchical clustering analysis of metabolites associated with (**C**) methionine and (**D**) tryptophan metabolism.

To further characterize the effects of HDA treatment on the biological metabolites in macrophages, MSEA was performed for metabolite enrichment analysis. The MSEA results revealed that metabolites significantly associated with methionine metabolism (*p* = 3.83 × 10^−5^), glycine and serine metabolism (*p* = 1.03 × 10^−4^), and tryptophan metabolism (*p* = 3.68 × 10^−3^) in positive ion mode (Figure 2A). In addition, metabolites were enriched in steroidogenesis (*p* = 1.26 × 10^−2^), nucleotide sugars metabolism (*p* = 1.95 × 10^−2^), and tyrosine metabolism (*p* = 2.60 × 10^−2^) under negative ion mode (Figure 2B). Among them, the betaine, L-cystathionine, and L-serine metabolites were found to be related to methionine metabolism (Figure 2C), whereas L-glutamic acid, L-tryptophan, and 5-hydroxytryptophan were related to tryptophan metabolism (Figure 2D).

### 2.3. Genes Alteration and Function Analysis Associated with 10-HDA Treated

**Figure 3:**
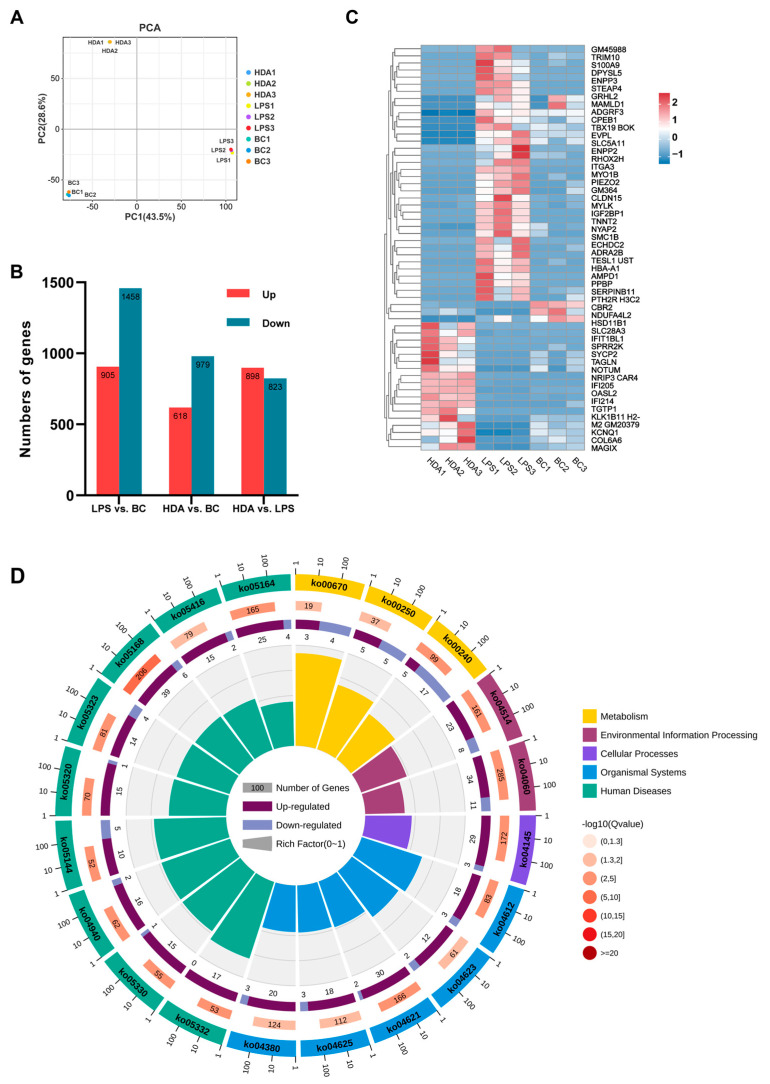
Identification and enrichment of coding transcripts (mRNA) expression. (**A**) PCA plot of all sample transcriptomes under the HDA, LPS treatments, and the control; (**B**) differential expressed genes statistics in macrophages with different treatments; (**C**) heatmap visualization of differentially expressed genes between the LPS and HDA groups (|fold change| ≥ 5) and the levels of the corresponding genes in the BC group; (**D**) KEGG pathway annotation of the DEGs between the LPS and HDA groups.

As shown in Figure 3A, the PCA score plot of the gene expression demonstrated three distinct clusters, which revealed the close proximity of the biological replicates. Differentially expressed genes (DEGs) were identified according to the cell treatment. As a result, 2363, 1597, and 1721 DEGs were identified among LPS vs. BC, HDA vs. BC, and HDA vs. LPS comparisons, respectively (Figure 3B). A heatmap illustrated clusters of transcripts with significant differential expression patterns (|fold change| ≥ 5) between HDA- and LPS-treated groups and the corresponding gene expression levels in the control group (Figure 3C). These DEGs were significantly enriched for immune system-associated KEGG categories such as antigen processing and presentation (ko04612), NOD-like receptor signaling pathway (ko04621), cytosolic DNA-sensing pathway (ko04623), and C-type lectin receptor signaling pathway (ko04625) (Figure 3D). Meanwhile, the DEGs were enriched in amino acid metabolism, including alanine, aspartate, and glutamate metabolism (ko00250), arginine biosynthesis (ko00220), and cysteine and methionine metabolism (ko00270), which was similar to the previous pathways of metabolic enrichment.

### 2.4. Combined Analysis of the Mechanism of LPS-Stimulated RAW 264.7 Cells Treated with 10-HDA

**Figure 4:**
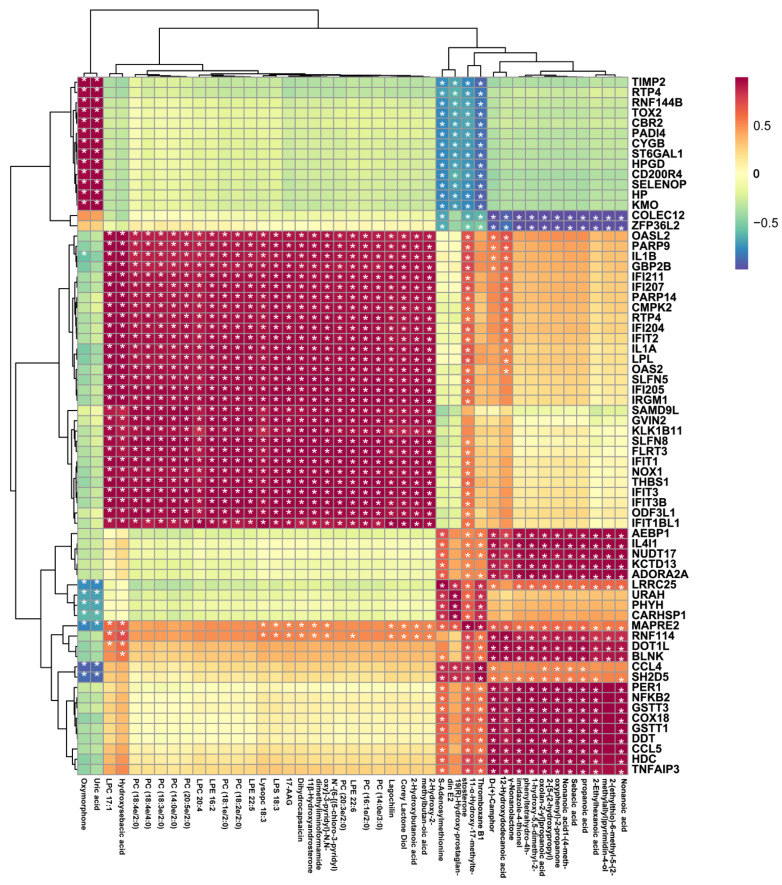
Heatmap map of correlation between DEG and DEM abundance. The asterisk indicates significant correlation, * *p* < 0.05.

To further explore the potential relationship between DEGs and DEMs, the metabolites and transcripts were Pearson correlation coefficients. Correlation analysis revealed that PC (20:5e/2:0), PC (14:0e/2:0), PC (18:3e/2:0), PC (18:4e/4:0) were significantly positively correlated with the expression of IL-1α (Interleukin 1α) and IL-1β (Interleukin 1β), while s-adenosylmethionine (SAM) levels were significantly negatively correlated with TOX2 (TOX high mobility group box family member 2) and CYGB (cytoglobin) expression, and positively correlated with the expression of IL4-i1 (interleukin 4 induced 1) (Figure 4). These findings suggest that glycerophospholipid metabolism and s-adenosylmethionine-dependent methylation processes may play important roles in the immunomodulatory effects of 10-HDA in macrophages. 

### 2.5. Quantitative Real-PCR Validation

**Figure 5:**
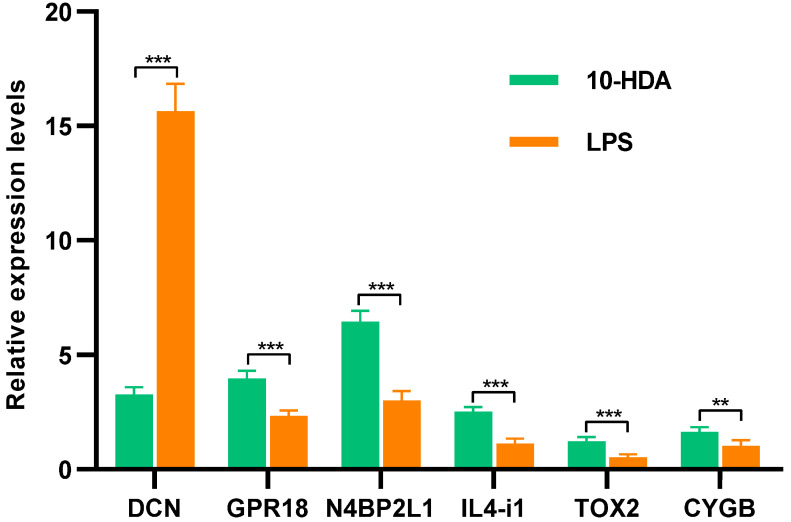
Relative expression levels of DEGs between HDA-treated cells and LPS-induced cells. The expression data are presented as mean ± SD. ** *p* < 0.01, *** *p* < 0.001.

To validate the RNA-seq results, three random DEGs (DCN, GPR18, and N4BP2L1) in differential analysis and three key genes (IL4-i1, TOX2, and CYGB) in correlation analysis were subjected to qPCR. As shown in Figure 5, N4BP2L1, GPR18, IL4-i1, CYGB, and TOX2 demonstrated significantly higher expression in HDA-treated cells than in LPS-induced cells, and the expression level of DCN significantly decreased with HDA treatment, which was consistent with the RNA-seq data. These results indicated that the RNA-seq data for the mRNAs were reliable. 

## 3. Discussion

Inflammatory macrophages play a critical role in immune responses, and their dysregulated metabolism can contribute to the development of various diseases [17]. Given their central role in inflammation and disease, macrophages represent an attractive therapeutic target. Targeting specific cytokines or chemokines that macrophages produce or inhibiting macrophage activation directly are two approaches that have shown promise in treating inflammatory diseases [18]. This study aimed to investigate the effects of 10-hydroxy-2-decenoic acid (10-HDA) treatment on the metabolism and transcriptome of inflammatory macrophages. Through the utilization of LC-MS/MS analysis in positive and negative ion modes and high throughput RNA sequencing, intracellular metabolites, and mRNAs were examined, and their profiles were compared between different treatment groups. The observed metabolic alterations in 10-HDA-treated inflammatory macrophages suggest the potential therapeutic effects of 10-HDA in modulating macrophage metabolism. The upregulation of specific metabolites, including PC (16:1e/2:0), PC (18:2e/2:0), and L-glutathione, indicates enhanced antioxidant capacity and cellular membrane stability [19,20]. L-Glutathione plays a pivotal role in maintaining redox balance within cells by serving as a potent antioxidant and protecting against oxidative stress-induced damage. The upregulated expression of L-glutathione has been associated with improved outcomes and enhanced cellular protection in certain diseases accompanied by alterations in other metabolites involved in redox balance, lipid metabolism, and energy homeostasis [21,22]. Moreover, the downregulation of metabolites such as methionine, L-tyrosine, and ornithine in 10-HDA-treated macrophages highlights the modulation of key metabolic pathways involved in inflammation and immune response [23,24]. These results suggested that 10-HDA treatment led to significant metabolic alterations in LPS-induced macrophages and its potential as a therapeutic agent in inflammatory diseases.

Metabolic reprogramming in macrophages is tightly linked to their functional states and immune responses [25]. The results of the MSEA analysis revealed significant associations between HDA treatment and specific metabolic pathways in macrophages. Our results showed that methionine metabolism and tryptophan metabolism were the most significantly enriched pathways in both ion modes, which are critical for macrophage function and immune responses [26]. Methionine is an essential amino acid and acts as a precursor for various metabolites, including s-adenosylmethionine (SAM), a key methyl donor in cellular processes [27]. The downregulation of methionine and the upregulation of metabolites involved in methionine metabolism, such as betaine and L-cysteine, indicate the potential modulation of these pathways via HDA treatment. The enrichment of metabolites related to tryptophan metabolism was consistent with the metabolic reprogramming of LPS-stimulated human lung macrophages that involved tryptophan metabolism [28]. The importance of tryptophan metabolism in macrophage function has been documented in previous studies. For instance, the upregulation of tryptophan metabolism-related metabolites in macrophages may indicate increased activity of the enzyme indoleamine 2,3-dioxygenase and subsequent production of kynurenine metabolites, which have been implicated in regulating immune responses, including the modulation of T cell function and the generation of immunosuppressive or neuroactive metabolites [29,30]. HDA has been reported to possess anti-inflammatory properties [15], and the modulation of these specific metabolic pathways may contribute to its therapeutic benefits.

Transcriptomic analysis revealed significant changes in gene expression patterns and pathway enrichment in macrophages upon HDA treatment. Antigen processing and presentation are essential for the recognition and activation of the adaptive immune response. The upregulation of DEGs associated with antigen-processing machinery, including proteasomal components, chaperones, and transporters, indicates an enhanced ability of macrophages to process and present antigens effectively [31]. Arginine serves as a substrate for nitric oxide synthase (NOS), leading to the production of NO, an important signaling molecule involved in inflammation and immune responses [32]. Furthermore, DEGs were enriched in the cytosolic DNA-sensing pathway NOD-like receptor (NLR) signaling pathway, and C-type lectin receptor (CLR) signaling pathway, suggesting its impact on macrophage function and phenotype [33]. Additionally, the NLR and CLR signaling pathways are interconnected with other immune signaling pathways, such as Toll-like receptor (TLR) signaling and inflammasome activation [34]. Crosstalk between these pathways allows for synergistic or antagonistic effects on macrophage responses, shaping the overall immune response to pathogens [35]. Our transcriptomic analysis revealed a significant regulation of genes involved in antigen processing, presentation, and amino acid, suggesting their impact on macrophage-mediated immune functions.

The interaction between gene expression and metabolite profiles plays a crucial role in understanding cellular processes and identifying potential therapeutic targets. The correlation analysis revealed potential associations between specific phosphatidylcholine metabolites and the expression of IL-1α and IL-1β, suggesting their involvement in the inflammatory response. Phosphatidylcholines are important components of cellular membranes and are known to participate in signal transduction and inflammation [36,37]. Moreover, the negative correlations between s-adenosylmethionine levels and the expression of TOX2 and CYGB, along with the positive correlation with IL4-il, indicate potential links between s-adenosylmethionine-dependent methylation processes and the modulation of gene expression in response to 10-HDA treatment. A previous study has shown that s-adenosylmethionine can reduce the pro-inflammatory cytokine TNF-α and increase the anti-inflammatory cytokine IL-10 in LPS-induced macrophages in association with changes in specific gene promoter DNA methylation [38]. The downregulation of CYGB-induced NO release in the presence of LPS and controlled LPS-induced inflammation by regulating macrophage response [39]. Together, the correlation analysis revealed potential associations between specific phosphatidylcholine metabolites and the expression of IL-1α and IL-1β, suggesting their involvement in the inflammatory response [40].

Previous studies have shown that 10-HDA (0–100 μM) inhibits the proliferation of normal human cells such as IMR90 lung fibroblasts, L-02 liver cells, and GES-1 gastric cells in time- and concentration-dependent manners, but no cytotoxicity was observed at low concentrations [41]. Additionally, treatment with 10-HDA in a certain concentration range does not cause toxicity to RAW264.7 cells, indicating the potential treatment of inflammation [16,42]. There are few studies on the toxic effects of 10-HDA on animals. Albalawi et al. have demonstrated that oral administration of 10-HDA at doses of 2.5 and 5 mg/kg/day for 14 days has no significant toxic effect on female Swiss albino mice [43]. A study revealed that the administration of 10-HDA into aged male Sprague Dawley rats (12–24 mg/kg/day) led to better maintenance of body weight [44]. Consequently, considering the toxicity effects of 10-HDA, further investigations are needed, particularly in the in vivo models.

## 4. Materials and Methods

### 4.1. Chemicals and Reagents

*Trans*-10-hydroxy-2-decenoic acid (10-HDA) and lipopolysaccharide (LPS) were purchased from Sigma (St. Louis, MO, USA). Dulbecco’s modified Eagle’s medium (DMEM), fetal bovine serum (FBS), methyl sulfoxide (DMSO), and TRIzol reagent were purchased from Thermo Fisher Scientific (Carlsbad, CA, USA). Penicillin, streptomycin, and other chemicals of analytical grade were purchased from Sangon Biotechnology. Co. Ltd. (Shanghai, China).

### 4.2. Cell Culture and Treatment

Murine RAW 264.7 macrophage cells were acquired from the Cell Bank of Chinese Academy of Sciences (Shanghai, China) and cultured in DMEM supplemented with 10% (*v*/*v*) fetal bovine serum, 1.5 g/L NaHCO_3_, and 1% penicillin-streptomycin solution. The cells were maintained in a humidified incubator with 5% carbon dioxide at 37 °C. A total of 5 × 10^3^ cells (100 μL/well) in the logarithmic phase of growth were seeded in each well of 96-well plates. RAW 264.7 cells (1 × 10^5^) were pretreated with 10-HDA (10 μg/mL) for 2 h and then stimulated with 1 μg/mL LPS and cultured for 24 h in 6-well plates. 

### 4.3. Metabolite Extraction

After removing the cell growth medium, cells were washed with sterile PBS. Cells were placed in EP tubes and resuspended in 300 μL of prechilled 80% methanol by well vortex. The samples were whirled for 30 s and sonicated for 6 min on ice. Then, they were centrifuged at 5000 rpm for 1 min at 4 °C, and supernatant was freeze-dried and dissolved with LC-MS-grade water. Finally, 200 μL of the supernatant was injected into the UHPLC-MS/MS system analysis. In total, 10 μL of the supernatant from each sample was pooled as a quality control (QC) sample and injected into analytical run at every five cell samples during the whole intervals.

### 4.4. UHPLC-MS/MS and Metabolites Analyses

UHPLC-MS/MS analyses were performed using a Vanquish UHPLC system (Thermo Fisher, Germering, Germany) with a Hypesil God column (2.1 nm × 100 nm, 1.9 μm) and coupled with an Orbitrap Q ExactiveTM HF-X mass spectrometer (Thermo Fisher, Germany) operating in positive/negative polarity mode. Raw data were generated using UHPLC-MS/MS and processed via Compound Discoverer 3.1 (Thermo Fisher, USA) for peak alignment, peak picking, and metabolite quantitation. The low-quality metabolite features were removed when 50% of the metabolite features in all samples or 30% of the QC samples cannot be detected. The main parameters of Compound Discoverer were set as follows: retention time tolerance, 2 min; actual mass tolerance, 5 ppm; signal intensity tolerance, 30%; signal/noise ratio, 3; and minimum intensity, 100,000. Peak intensities were normalized to the total spectral intensity for the molecular formula prediction based on additive ions, molecular ion peaks, and fragment ions. The accurate qualitative and relative quantitative results of metabolites were obtained by peaks matching against the mzCloud (https://www.mzcloud.org/, accessed on 3 November 2021) and mz Vault and Mass List database.

Principal component analysis (PCA) and partial least squares discriminant analysis (PLS-DA) was performed with the R package ropls for multivariate statistical analysis. A variable importance in the projection (VIP) value based on orthogonal projections to latent structures discriminant analysis (OPLS-DA) was applied to rank the metabolites [45]. For analyses of differential metabolite profiles, a VIP value ≥ 1, *p* value of Student’s *t* test < 0.05, and fold change > 2 were set as the thresholds for the identification of differential metabolites. The metabolic pathway annotation analysis of differential metabolites was performed using the Kyoto Encyclopedia of Genes and Genomes (KEGG) database. Metabolic set enrichment analysis (MSEA) was performed with MetaboAnalyst R package (https://rdrr.io/github/afukushima/MSEAp/, accessed on 3 November 2021) to evaluate for pathway over-representation [46].

### 4.5. UHPLC-MS/MS and Metabolites Analyses 

Total RNA was isolated from the cells in each group using TRIzol reagent kit according to the manufacturer’s protocol. RNA purity and integrity were assessed on a Bioanalyzer 2100 system (Agilent, Santa Clara, CA, USA) and RNase-free agarose gel electrophoresis. A total of 3 μg RNA enriched using Oligo (dT) beads per sample was reversely transcribed into cDNA using NEBNext^®^ Ultra^TM^ RNA Library Prep Kit for Illumina following the manufacturer’s instructions. After cluster generation using the TruSeq PE Cluster Kit v3-cBot-HS (Illumina, San Diego, CA, USA), the cDNA libraries were sequenced on the Illumina Novaseq 6000.

### 4.6. Gene Differential Expression and Enrichment Analyses

Clean reads were obtained after removing reads containing adapters, more than 10% of unknown nucleotides, and reads with low-quality (Q-value ≤ 20) bases. Then, short reads that aligned to the ribosome RNA database with Bowtie2 v.2.2.8 were removed [47]. The remaining clean reads were mapped to the reference genome (mm10) using Hisat2 v2.4 [48] and assembled via StringTie v1.3.1 [49] using the default parameters. The expression abundance and variations of mRNA were calculated as fragments per kilobase of transcript per million mapped reads (FPKM) using RSEM software [50]. Differential expression analysis was performed using DESeq2 [51], and a false discovery rate (FDR) < 0.05 and |fold change| ≥ 2 were considered as the thresholds of significant differential expression.

### 4.7. Gene Differential Expression and Enrichment Analyses 

In order to integrate the transcriptomic and metabolomic data, gene and metabolite pairs were ranked in descending order of absolute Pearson correlation coefficients [52]. The top pairs of genes and metabolites with an absolute Pearson correlation > 0.5 were applied for metabolite transcript network analysis.

### 4.8. Real-Time Quantitative Polymerase Chain Reaction Analysis

Total RNA was isolated from the cells using TRIzol reagent and reverse transcribed into first-strand cDNA with the PrimeScript™ RT reagent Kit with gDNA Eraser. Real-time quantitative PCR was performed on an ABI 7500 Real-Time PCR system (Applied Biosystems, Foster City, USA) with specific primers (Table 1) in the following conditions: 95 °C for 30 s, 40 cycles of 95 °C for 5 s and 60 °C for 34 s, and subsequent melting curve analysis. The differences in the relative expression levels of genes were analyzed using an independent-samples t-test with SPSS 22.0 software (IBM, Armonk, NY, USA) based on the GAPDH expression levels using the 2^−ΔΔCt^ method [53] with three independent biological replicates.

## 5. Conclusions

In this study, we investigated the metabolite and mRNA profile changes associated with the anti-inflammatory effects of 10-HDA treatment on LPS-stimulated RAW 264.7 cells. Metabolite enrichment and DEG enrichment analyses indicated that amino-acid-related metabolism may be the major metabolic pathway treated with 10-HDA in RAW 264.7 cells. The results of combined analyses of RNA-seq and LC-MS/MS data strongly suggest that glycerophospholipid metabolism is closely related to the anti-inflammatory of 10-HDA. Our study provides insights into the molecular mechanism of 10-HDA in inflammatory macrophages and a basis for further research on therapeutic potential in inflammatory diseases.

## Figures and Tables

**Table 1:** The sequences of primers for the selected genes.

Gene	Forward (5′-3′)	Reserve (5′-3′)	bp
GAPDH	TGACCTCAACTACATGGTCTACA	CTTCCCATTCTCGGCCTTG	85
DCN	TCTTGGGCTGGACCATTTGAA	CATCGGTAGGGGCACATAGA	119
GPR18	CACCCTGAGCAATCACAACCA	AGTGACATTAACAAACAGCCCA	121
N4BP2L1	TATTCCGGGAACCCGACACT	GAAGGACACTGTGAAAGGTAACA	138
IL4-i1	AACACTTGTTGGTGGAAACGA	TCCTTGCGATTAGGAGTGGTC	241
TOX2	TATAACGCCTCCCAATCTCCC	TTGCTTGGTACGAGTAGGCAG	125
CYGB	GGCGACATGGAGATAGAGCG	CTCGCAGTTGGCATACAGC	102

## Data Availability

The data presented in this study are available upon request from the corresponding author.
